# The Effects of the Reverse Current Caused by the Series Compensation on the Current Differential Protection

**DOI:** 10.1155/2014/473913

**Published:** 2014-08-26

**Authors:** Cui Tang, Xianggen Yin, Xuanwei Qi, Zhe Zhang, Minghao Wen

**Affiliations:** State Key Laboratory of Advanced Electromagnetic Engineering and Technology, Huazhong University of Science and Technology, 1037 Luoyu Road, Wuhan 430074, China

## Abstract

The series capacitor compensation is one of the key technologies in the EHV and UHV long distance power transmission lines. This paper analyzes the operation characteristics of the main protection combined with the engineering practice when the transmission line overcompensation due to the series compensation system is modified and analyzes the influence of the transition resistance and the system operation mode on the current differential protection. According to the simulation results, it presents countermeasure on improving the sensitivity of differential current protection.

## 1. Introduction

With the rapid development of large-capacity transmission and smart grid, the series capacitor compensation technology has been increasingly used in ultrahigh voltage transmission line. Series capacitor compensation could improve the transmission capacity and stability of the power system and also bring a great effect on the protection [1–5]. For solving the problems of series compensated lines, relay protection workers do a lot of theoretical research and practice of inspection work [2–8] and also do the research on analysis of actual series compensation project protection [9–15]. The transmission line possible overcompensation due to the series compensation system is modified, it will bring huge challenges to the main protection and back-up protection.

The series compensation degree of transmission line K is usually described as the percentage of the capacitance value of the series compensation capacitor and reactance value for the transmission line. Series compensation degree is usually about 30% in the actual project; more than 40% belong to the high line series compensation degree. In addition, from the point of view of the influence on relay protection, it needs to consider the ratio of the capacitance value of the series capacitor compensation and the system equivalent impedance, which is different from the line series compensation degree, and also will affect the protection.

With the grid development and load growth, the system structure and the equivalent impedance of the system may be changed. For example, insert the new transformer substation on the both ends buses of the line; the equivalent impedance of the power system becomes smaller so that the proportion of the series capacitor compensation capacitance value relative to the system impedance increases, thus affecting the movement behavior of the protection. At present, there is a special case, namely, due to the system renovation or expansion, in the series compensated lines access a new substation, the original series capacitor compensation capacitive reactance does not change, so the new line compensation degree is exceed 50%, while also makes the series compensation system structure changed; the system operation mode increases. These situations will make the problem of voltage and the current reverse more serious and will adversely affect the main protection and back-up protection of the series compensated lines and adjacent lines or even reduce the current differential protection sensitivity. Therefore, how to research the effects of series capacitor compensation on protection after the grid structure or system parameters change and propose the improvement measures has a very important engineering significance for giving full play to the advantages of series compensated transmission line. This paper, combined with practical engineering, analyzes the effects of the reverse current which is induced by the series compensation on current differential protection in the series compensated degree transmission line and gives improvement measures on the problem of low sensitivity in internal faults.

## 2. Simulation Model of High Series Compensated Degree Transmission Line

### 2.1. Simulation Model of Grid Network

**Figure 1:**
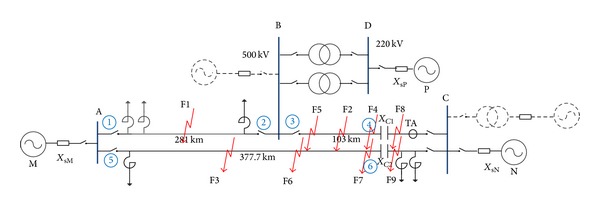
Simulation model of power grid.

Generally speaking, a new substation is installed on one end of the series compensation line (as the dotted line shown in Figure 1); this case is easier to understand; so, this will not be considered here. The simulation, combined with practical engineering, take the complex situation as an example, namely, a new transformer substation is inserted in the series compensated transmission line and considering the operation mode of system and series compensation degree of the transmission line increased, system network as the solid lines is shown in Figure 1. The new substation B built on the line ACI formed the line AB and line BC; the length of line AB is 281 km and the length of line BC is 103 km, because the series capacitor unchanged, causing the line BC series compensation to increase; the degree of compensation reached 174%. Using the electromagnetic transient simulation software to establish simulation model, the parameters in the model were set up according to the actual situation of the project; the parameters of transmission line are positive sequence impedance in per km *Z*_*L*1_ = *Z*_*L*2_ = (0.0242 + 0.295*j*) Ω/km, zero-sequence impedance in per km *Z*_*L*0_ = (0.299 + 1.33*j*) Ω/km, positive sequence capacitance in per km *C*_1_ = *C*_2_ = 0.01612 uF/km, and zero-sequence capacitance in per km *C*_0_ = 0.009441 uF/km. The equivalent parameters of power system are considered as the system in the large and small operation mode. The system parameters in the large operation mode: the positive sequence impedance of the equivalent source M (*X*_sM1_) is 12.9 Ω, the zero-sequence impedance of the equivalent source M (*X*_sM1_) is 13.75 Ω, the positive sequence impedance of the equivalent source N (*X*_sN1_) is 10.5 Ω, and the zero-sequence impedance of the equivalent source N (*X*_sN0_) is 29.75 Ω (the actual value in 500 KV); the positive sequence impedance of the equivalent source P (*X*_sP1_) is 117 Ω and the zero-sequence impedance of the equivalent source P (*X*_sP0_) is 43.56 Ω (the actual value in 220 KV). The system parameters in the small operation mode are as follows: *X*_sM1_ =* j*143 Ω, *X*_sM0_ =* j*57.25 Ω, *X*_sN1_ =* j*30 Ω, *X*_sN0_ =* j*57 Ω, *X*_sP1_ is infinity, and *X*_sP0_ =* j*43.56 Ω.

### 2.2. Simulation Model of Series Compensation System

The related structure and parameter of series compensation system are in accordance with the engineering actual situation. The compensation level of FSC is 30%, the reactance of FSC *X*_FSC_ = 33.43 Ω, and the compensation level of TCSC is 15%, the reactance of TCSC *X*_TCSC_ = 16.71 Ω. The simulation model of FSC considered the series compensation capacitor group, metal oxide varistor (MOV), the spark gap, the bypass circuit breaker, and its damping discharge circuit. The difference of the TCSC simulation model compared with FSC is the TCR branch. The* V-I* characteristic curve of MOV based on the actual series compensated device parameters.

### 2.3. Simulation Model of Protection

This paper analyzed the operating characteristics of current differential protection. The typical criterion of current differential protection is given as follows.

Segregated phase current differential protection equation is

(1) $$\begin{array}{c} I_{d}>I_{\text{set}},\;I_{d}>0.6I_{r}. \end{array}$$

Zero-sequence current differential protection equation is as follows:

(2) $$\begin{array}{c} I_{d0}>I_{\text{set}},\;I_{d0}>0.75I_{r0}. \end{array}$$

Fault component current differential protection equation is

(3) $$\begin{array}{c} \Delta I_{d}>I_{\text{set}},\;\Delta I_{d}>0.75\Delta I_{r}, \end{array}$$

where *I*_*d*_ is the differential current, $I_{d}=\left|{\dot{I}}_{m}+{\dot{I}}_{n}\right|$; *I*_*r*_ is the restraint current, $I_{r}=\left|{\dot{I}}_{m}-{\dot{I}}_{n}\right|$; ${\dot{I}}_{m}$,  ${\dot{I}}_{n}$ are, respectively, the current phasor at both ends of the transmission line; *I*_set_ is the setting value; *I*_*d*0_ is the zero-sequence differential current; *I*_*r*0_  is the zero-sequence restraint current; Δ*I*_*d*_ is the fault components differential current; and Δ*I*_*r*_ is the fault components restraint current.

## 3. The Fault Feature of the Transmission Line with High Series Compensation Degree

In the series compensation system, the relationship of the electric parameters may has fundamental change when the faults occur on the line, such as reverse current and reverse voltage. The greatest impact on current differential protection of the series compensated line is the problem of reverse current.

To examine the current reverse situation by analyzing the phase relationship between voltage and current on both sides of the line, current reference direction is from the measurement point behind the bus to the line side.

According to the actual operation situation, after line ACI inserts a new substation B, MOV high current protection setting value is unchanged; so, it allows MOV high current protection to operate at the external fault. The MOV high current protection setting value of FSC and TCSC is, respectively, 10.5 kA and 12.0 kA. According to the simulation, MOV high current protection of series compensation system in the line BC will operate when the three-phase fault (ABC) occurs at the fault points F2, F4, and F5; and it does not operate when single-phase grounding fault (AG) occurs with the transition resistance being greater than 150 Ω.

The system is in the larger operation mode; taking the fault point F4 as the example, when the three-phase short circuit occurs at the fault point F4, positive sequence voltage and current phase of measuring point ③ are 85.3; the measured impedance is inductive. MOV high current protection of series capacitor compensation will operate at this situation. The positive sequence measurement impedance at the back of measurement point ④ is also inductive so that the situation of reverse current does not occur.

The phase relationships of negative sequence and zero-sequence voltage and current are shown in Table 1 when AG occurs at the fault point F4.

MOV high current protection of series capacitor compensation does not move when single-phase grounding fault with high-impedance occurs at the fault point F4; so, the series capacitor operates in the system; the positive sequence impedance measurement at the back of measurement point ④ is also capacitive; it causes current to reverse.

## 4. Operation Situation of the Current Differential Protection

Current differential protection as the main protection of series compensated lines: its operating characteristics are the key point of this study. In this paper, take ABC and AG as an example to illustrate the operation features of current differential protection. For AG, consider the transition resistance is 0 Ω, 150 Ω, and 300 Ω, respectively. Electrical quantities are actual values on the primary side in this paper.

### 4.1. Operation Situations of Current Differential Protection on the Line AB

Take ABC and AG that occurred at the fault point F1 in large operation mode as an example. The simulation results are shown in Table 2.

When the high resistance grounding fault occurs at the fault point F1, the fault component current differential protection and zero-sequence current differential protection are correct to operate, but segregated phase current differential protection refuses to operate. In this situation, both sides of the line AB does not appear current reverse, segregated phase current differential protection refuses to operate because of insufficient sensitivity, illustrates that the fault component current differential protection and zero-sequence current differential protection with higher sensitivity and can operate correctly in the inductive system network.

### 4.2. Operation Situations of Current Differential Protection on the Line BC

Take ABC and AG that occurred at the fault point F4 in large operation mode as the example. The simulation results are shown in Table 3.

When the high resistance grounding fault occurs at the fault point F4, segregated phase current differential protection and fault component current differential protection refuse to operate. Zero-sequence current differential protection can be effective operation, but, at this time, the zero-sequence differential current decreases; its sensitivity is low.

## 5. Analysis of the Influence Factors on Current Differential Protection

Current differential protection of the series compensated line refuses to operate due to the lack of sensitivity; this issue is related to the series compensation degree, equivalent impedance and angle on both sides of the system, MOV characteristics and its high current protection of series compensation systems (including the FSC and TCSC), fault form (type and location), the transition resistance, and other factors. This paper examines the impact of transition resistance and system operation mode on current differential protection.

### 5.1. The Impact of Transition Resistance on Current Differential Protection

When a single-phase grounding fault occurs at the fault point F4, the protection operation situation is shown in Table 4 when the transition resistance is different. MOV high current protection operates when the transition resistance is 0 Ω; in the other situation, series compensation is serial in the system.

From Table 4, the transition resistance is 0, 20, 50, and 100 Ω, respectively; current differential protection and zero-sequence current differential protection can operate, but the sensitivity of current protection is low. When the transition resistance is greater than 20 Ω, fault component current differential protection may refuse to operate. So, the sensitivity of phase current differential protection and zero-sequence differential protection on the line BC should be greater.

### 5.2. The Impact of the Operation Mode on the Current Differential Protection

Equivalent impedance and mode of power flow changing trends will impact the current differential protection indeed. This study considered the effects of system operating modes and power angle on current differential protection.

After the system changed to a small operation mode, current differential protection can be correct and reliable operation when ground fault with 300 Ω impedance occurs at the different fault points. When the operation mode becomes smaller, the power flow of the system becomes smaller at normal operation, the restraint current before the fault occurred is becoming smaller, it superimposed with the fault component current, so the restraint current after the fault occurred is also small, the phase current differential protection can operate correctly.

The zero-sequence current differential protection is correct and reliable operation when grounding fault with 300 Ω impedance occurs in a series compensated line, regardless of the fact that the system is in the large or small operation mode. When the system is in a small operation mode, sensitivity of the zero-sequence current differential protection increased; zero-sequence differential current is greater than zero-sequence current brake; the protection can ensure operation when the fault at the exit of series compensated on the line BC.

**Figure 2:**
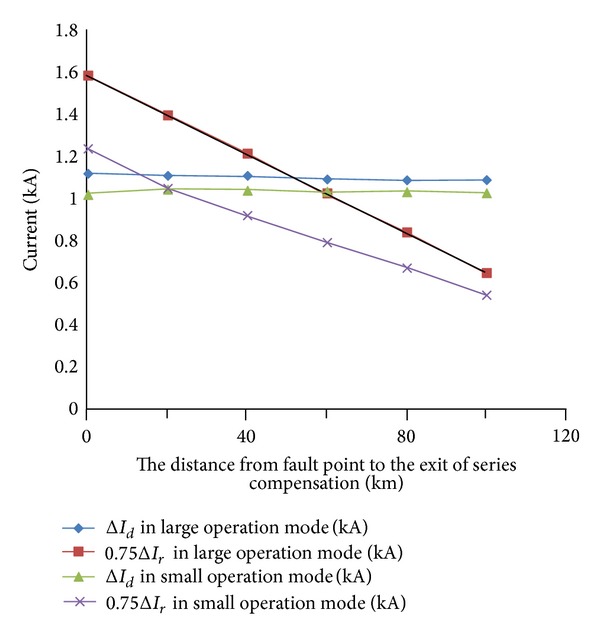
The relationships of Δ*I*_*d*_ and 0.75Δ*I*_*r*_ when the single phase grounding fault with 300 Ω transition resistance occurred at different places on the line BC.

When AG (300 Ω) impedance occurs at different distances from the exit of series compensation on the line BC, relationships of Δ*I*_*d*_ and 0.75Δ*I*_*r*_ on the line BC are shown in Figure 2.

According to Figure 2 and formula (3), the system is in the large operation mode; the fault component current differential protection can operate when the fault distance is 50 km from the exit of series compensation. When the system is in the small operation mode, the fault component current differential protection can operate when the fault distance is 20 km from the exit of series compensation line.

## 6. Measures for Improving the Current Differential Protection

From the simulation results, with the changes of network structure and expansion of the system, segregated phase current differential protection refused to operate due to insufficient sensitivity because the fault current is small when the high resistance grounding fault occurs at the series compensation transmission line. Even the sensitivity of zero-sequence current differential protection can satisfy the requirements, but, in the engineering applications, the operation speed of segregated phase current differential protection is faster. Therefore, in order to improve the speed and sensitivity of the overall protection, it is necessary to improve the sensitivity of phase current differential protection in order to accelerate the movement speed of the protection.

### 6.1. Using Three Ratio Restraint Characteristic Curves

**Figure 3:**
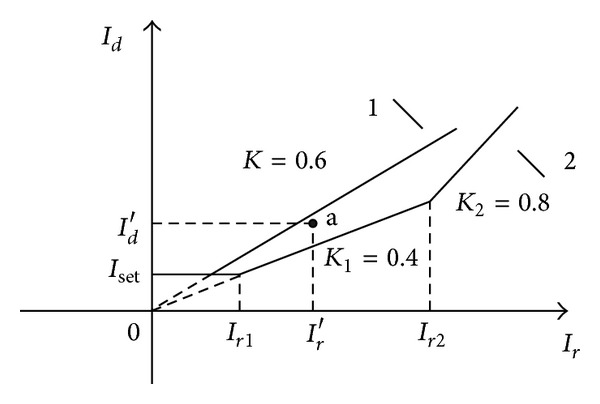
The characteristic curves of segregated phase current differential protection.

Current differential protection using the three ratio restraint characteristics can improve protection performance and avoid protection refusing to operate at internal fault or maloperation at external fault. If the criterion of segregated phase current differential protection is [16, 17]:

(4) $$\begin{array}{c} I_{d}>I_{\text{set}}\;\left(0\leq I_{r}\leq I_{r1}\right),\\ I_{d}>K_{1}I_{r}\;\left(I_{r1}<I_{r}\leq I_{r2}\right),\\ I_{d}>K_{2}\left(I_{r}-I_{r2}\right)+K_{1}I_{r2}\;\left(I_{r2}<I_{r}\right), \end{array}$$

where *I*_set_ is the setting value of segregated current differential protection, *K*_1_ and *K*_2_ is the restraint factor. According to the engineering application, take *K*_1_ = 0.4, *K*_2_ = 0.8, the characteristic curve of protection as the curve 2 is shown in Figure 3. The original characteristic curve of protection as the curve 1 is shown in Figure 3. Segregated phase current differential protection can operate when AG (300 Ω) occurs at the fault point F4, as the point a in Figure 3.

### 6.2. Reducing the Ratio Restraint Coefficient of the Current Differential Protection

Restraint current in this simulation model of current differential protection is $I_{r}=\left|{\dot{I}}_{m}-{\dot{I}}_{n}\right|$, and restraint current in some other typical protection is $\left|{\dot{I}}_{m}-{\dot{I}}_{n}\right|/2$ [18]. The operating experience shows that the latter has been fully able to satisfy the requirements of reliable not malfunction when external fault occurred. Therefore, could consider using the latter form as the restraint current, or appropriate to reduce the restraint coefficient* K* when using the former form, which can increase the sensitivity of the line current differential protection. From the simulation, when AG (300 Ω) occurs at the fault point F1 or F4, the current differential protection can operate when using this measure.

It can be seen that reducing the restraint coefficient ratio of current differential protection in the transmission lines with high series compensated degree could improve the sensitivity of protection.

## 7. Conclusions

On the series compensation transmission line, there are many factors affected current differential protection, made it refuse to operate due to insufficient sensitivity. In this paper, combined with a project, mainly analyzes the effect of transition resistance and operation mode on the current differential protection. According to the research and analysis, get the following conclusions:

1. Single-phase grounding fault with transition resistance occurs in the series compensated lines; segregated phase current differential protection may refuse to operate. The zero-sequence current differential protection can have a sensitive and reliable operation; its sensitivity is higher.
2. When the system operation mode becomes larger, the reverse current is the more likely to occur, the impact on the current differential protection is the larger; conversely, when the system operating mode decreases, the sensitivity of current differential protection improves.
3. This paper puts forward the improvement of current differential protection ideas under the following project application conditions: using of multisegment ratio characteristic curve and reasonable reducing of the ratio of the current differential protection restraint coefficient. These improvements will also help to improve the sensitivity of phase selector in zero-sequence current differential protection.

## Figures and Tables

**Table 1:** The phase relationships of negative sequence and zero-sequence voltage and current when AG occurred at F4.

Fault type	③		④	
	Negative sequence/(°)	Zero-sequence/(°)	Negative sequence/(°)	Zero-sequence/(°)
AG (0 Ω)	−92.5	−82.7	−91.9	−100.1
AG (150 Ω)	−94.1	−93.7	91.3	102.6
AG (300 Ω)	−94.1	−93.7	91.3	102.6

**Table 2:** Operation of current differential protection when the different fault occurred at the fault point F1.

Fault type	Segregated phase current differential protection	Fault component current differential protection	Zero-sequence current differential protection
ABC (0 Ω)	*✓*	*✓*	*✓*
AG (0 Ω)	*✓*	*✓*	*✓*
AG (150 Ω)	*✓*	*✓*	*✓*
AG (300 Ω)	*✗*	*✓*	*✓*

**Table 3:** Operation of current differential protection when the different fault occurred at the fault point F4.

Fault type	Segregated phase current differential protection	Fault component current differential protection	Zero-sequence current differential protection
ABC (0 Ω)	*✓*	*✓*	*✓*
AG (0 Ω)	*✓*	*✓*	*✓*
AG (150 Ω)	*✓*	*✗*	*✓*
AG (300 Ω)	*✗*	*✗*	*✓*

**Table 4:** Operation of current differential protection on the different ground fault situation at the fault point F4.

Transition resistance/Ω	Segregated phase current differential protection	Fault component current differential protection	Zero-sequence current differential protection
0	*✓*	*✓*	*✓*
20	*✓*	*✗*	*✓*
50	*✓*	*✗*	*✓*
100	*✓*	*✗*	*✓*
